# Use of Zebrafish to Probe the Divergent Virulence Potentials and Toxin Requirements of Extraintestinal Pathogenic *Escherichia coli*

**DOI:** 10.1371/journal.ppat.1000697

**Published:** 2009-12-18

**Authors:** Travis J. Wiles, Jean M. Bower, Michael J. Redd, Matthew A. Mulvey

**Affiliations:** 1 Division of Cell Biology and Immunology, Department of Pathology, University of Utah, Salt Lake City, Utah, United States of America; 2 Huntsman Cancer Institute, University of Utah, Salt Lake City, Utah, United States of America; University of Washington, United States of America

## Abstract

Extraintestinal pathogenic *E. coli* (ExPEC) cause an array of diseases, including sepsis, neonatal meningitis, and urinary tract infections. Many putative virulence factors that might modulate ExPEC pathogenesis have been identified through sequencing efforts, epidemiology, and gene expression profiling, but few of these genes have been assigned clearly defined functional roles during infection. Using zebrafish embryos as surrogate hosts, we have developed a model system with the ability to resolve diverse virulence phenotypes and niche-specific restrictions among closely related ExPEC isolates during either localized or systemic infections. In side-by-side comparisons of prototypic ExPEC isolates, we observed an unexpectedly high degree of phenotypic diversity that is not readily apparent using more traditional animal hosts. In particular, the capacity of different ExPEC isolates to persist and multiply within the zebrafish host and cause disease was shown to be variably dependent upon two secreted toxins, α-hemolysin and cytotoxic necrotizing factor. Both of these toxins appear to function primarily in the neutralization of phagocytes, which are recruited in high numbers to sites of infection where they act as an essential host defense against ExPEC as well as less virulent *E. coli* strains. These results establish zebrafish as a valuable tool for the elucidation and functional analysis of both ExPEC virulence factors and host defense mechanisms.

## Author Summary

*Escherichia coli* can exist among the normal intestinal microbiota without causing any overt problems for the human host. However, humans as well as other animals can often acquire less-mild mannered variants of *E. coli* strains known as extraintestinal pathogenic *E. coli* (ExPEC) that can colonize sites outside of the intestinal tract and cause a range of serious illnesses, including sepsis, meningitis, and urinary tract infections. Despite many advances over the years using cell culture and rodent infection models, the spectrum of genes that control the ability of different ExPEC strains to colonize and grow within specific host niches and cause disease remain, for the most part, elusive. Here, we report the development of a new model system that uses zebrafish as surrogate hosts for ExPEC and related isolates. Using zebrafish to model both localized and systemic infections, we found that closely related ExPEC isolates display an unexpected array of virulence characteristics and toxin requirements that are not readily apparent from genomic information alone. This model system is amenable to high-throughput genetic and pharmacological screens and should prove useful in the development of more efficacious therapeutics.

## Introduction

*Escherichia coli* is a laboratory workhorse that has helped expand our fundamental understanding of many biological processes. Outside the laboratory, *E. coli* are a remarkably diverse species both genetically and with respect to their ability to exist as either harmless commensals or as pathogens in a variety of animal hosts. Substantial morbidity and annual medical costs in the billions of dollars are attributed to a group of *E. coli* strains referred to as extraintestinal pathogenic *E. coli* (ExPEC) [1],[2],[3],[4]. These pathogens have the capacity to persist within the human gut among the normal microbiota without any overt consequences. However, once outside the intestinal tract, ExPEC pathotypes can cause an array of diseases including urinary tract infections (UTIs), sepsis, and meningitis. The frequency of ExPEC-induced infections in human populations may be aggravated by the broad host range of these pathogens. For example, production birds raised for human consumption are susceptible to colibacillosis, a lethal infection caused by ExPEC strains known as avian pathogenic *E. coli* (APEC). These pathogens are highly similar to ExPEC strains like uropathogenic *E. coli* (UPEC), which are the primary cause of UTIs in humans [5],[6]. Such observations highlight the zoonotic potential of APEC and related bacteria, suggesting that the widespread dissemination of ExPEC-associated virulence traits among human isolates may occur through consumption of contaminated poultry or other food products [6],[7],[8].

In recent years, an enormous amount of information has been accrued by sequencing the genomes of several prototypic ExPEC isolates and other *E. coli* strains. These data, together with epidemiological analyses, confirm that distinct ExPEC pathotypes share many known and putative virulence factors. These include a number of secreted toxins, iron acquisition systems (siderophores), adhesins, and capsular antigens [9]. Secreted toxins, which include α-hemolysin (HlyA), cytotoxic necrotizing factor-1 (CNF1), and the secreted autotransporter SAT, can alter host signaling cascades, disrupt inflammatory responses, and induce host cell death, while siderophores like aerobactin, bacteriocin, and enterobactin allow ExPEC to sequester essential iron away from the host [10],[11],[12]. Adhesive organelles such as type 1, F1C, P, and S pili (or fimbriae) can mediate ExPEC interactions with, and entry into, host cells and tissues, while capsule expression may enable ExPEC to better avoid professional phagocytes [9],[13],[14]. These and other virulence factors are often encoded within genomic regions, known as pathogenicity islands (PAIs), which are acquired by horizontal gene transfer [15],[16],[17],[18].

The modular exchange of PAIs and other genetic elements likely provides ExPEC with a high degree of versatility over time, enabling these bacteria to adapt to and colonize assorted host and environmental niches. While an individual ExPEC isolate will typically encode about 5,000 genes, the total number of genes that may be swapped among all ExPEC isolates and related strains in nature is currently estimated to total more than 17,800 [19],[20]. However, most of these genes remain uncharacterized and factors that dictate the host and niche specificities of distinct ExPEC isolates remain enigmatic. Complicating matters further are results showing that even relatively well-studied virulence factors such as HlyA and CNF1, which are closely linked with UPEC and other ExPEC pathotypes, have effects on bacterial colonization and persistence that can be difficult to discern using established mouse infection models (unpublished observations and [21],[22],[23],[24]). Such observations, coupled with the sheer number of genes that ExPEC isolates can potentially acquire, indicate that these pathogens have likely evolved multiple and probably redundant mechanisms to overcome the many challenges encountered within diverse host environments.

To formally address this possibility, and to establish a more experimentally amenable high-throughput approach to phenotypically assess ExPEC virulence potentials during both systemic and localized infections, we developed a novel model system employing zebrafish (*Danio rerio*) embryos. Results obtained using this surrogate host model demonstrate that even very closely related ExPEC isolates can differ greatly in their virulence capacity in a niche- and dose-specific fashion. In addition, we found that the *in vivo* survival and virulence of different ExPEC isolates can be variably dependent upon the secreted toxins HlyA and CNF1, which appear to act primarily in the neutralization of host phagocytes. In total, this work highlights the phenotypic diversity of closely related ExPEC isolates and establishes zebrafish as a valuable tool for the discovery and analysis of both ExPEC virulence factors and key host defense mechanisms.

## Results

### Localized and systemic ExPEC infections in zebrafish

The pathogenic potential of ExPEC isolates has generally been assessed *in vivo* using rodent models in which bacteria are inoculated via either trans-urethral catheterization or other injection routes. These model systems mimic many important aspects of both localized UTIs and the often more serious systemic infections caused by ExPEC. However, these models do not lend themselves readily to rapid high-throughput analyses or real-time observations of the infection process. Over the past several years, zebrafish have emerged as a powerful vertebrate model system for deciphering virulence mechanisms employed by both fish-specific and mammalian pathogens [25],[26],[27],[28],[29],[30],[31]. At 48 h post fertilization (hpf) the zebrafish immune system is composed solely of innate defenses, including antimicrobial peptides, complement, toll-like receptors, and phagocytes [32],[33],[34],[35]. This developmental state makes zebrafish embryos a convenient model organism to study ExPEC pathogenesis since immunity to these pathogens in both humans and mice is also primarily dependent upon innate defenses [36]. Furthermore, the transparency and external development of zebrafish embryos facilitate the direct observation of pathogenic events in live animals [37]. To test the utility of zebrafish as an alternative host for ExPEC, we inoculated 48 hpf embryos with bacteria at two sites: a fluid-filled sac surrounding the heart referred to as the pericardial cavity and the blood, via the circulation valley (**Figure S1**). The pericardial cavity is an infection site that restricts bacterial dissemination and serves as a model for a localized infection [38]. Inoculation into the circulation valley results in the rapid dispersion of bacteria throughout the embryo, mimicking a systemic infection. Each site likely presents ExPEC with different challenges in terms of nutrient availability, phagocyte numbers, and the types and levels of antimicrobial agents.

To address the phenotypic diversity of ExPEC *in vivo*, we examined seven fully sequenced ExPEC isolates: two cystitis strains (UTI89 and F11), two pyelonephritis isolates (CFT073 and 536), one neonatal meningitis *E. coli* (NMEC) strain (RS218), an APEC isolate (APEC01), and one strain (83972) responsible for asymptomatic bacteruria (ABU). ABU isolates like 83972 can exist in a commensal-like relationship with the host, and in some cases appear to protect the urinary tract from colonization by more pathogenic bacterial strains [39]. We also utilized a recently sequenced human gut isolate (HS) and two laboratory K12 reference strains (MG1655 and W3110) as controls. Bacteria were grown in M9 minimal medium and washed in phosphate buffered saline (PBS) prior to injection into either the pericardial cavity or blood of 48 hpf zebrafish embryos (**Videos S1** and **S2**). Low (2,000 to 3,500 CFU), medium (4,000 to 6,500 CFU), and high (6,600 to 11,500 CFU) inoculation doses were used. Infected embryos were examined every 6 h over a 24 h period, and death was scored as the absence of a heartbeat and lack of blood flow.

**Figure 1:**
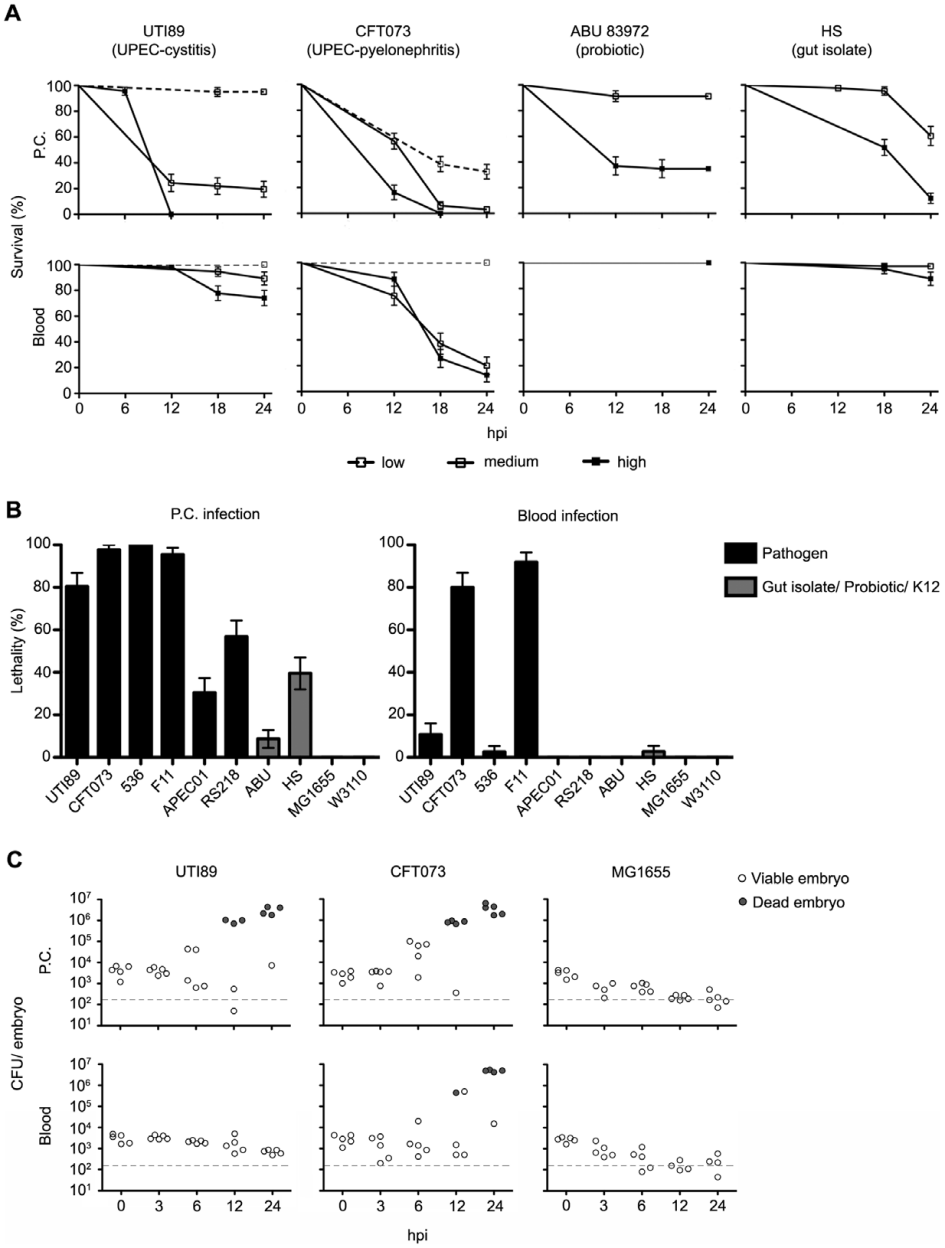
Zebrafish survival following infection with ExPEC and related strains. (A) The pericardial cavities (P.C., top row) or blood (bottom) of 48 hpf embryos were inoculated with PBS containing UTI89, CFT073, ABU 83972, or HS at low (2,000 to 3,500 CFU), medium (4,000 to 6,500 CFU), or high (6,600 to 11,500 CFU) doses. Fish were scored for death at 0, 6, 12, 18, and 24 h post-inoculation (hpi). Data are presented as Kaplan-Meier survival plots with standard error calculated by the Greenwood method. Absence of a tick mark indicates that no deaths were observed at that given time point. (B) Bars show percentage (±standard error) of fish that were killed by ∼5,000 CFU of each strain by 24 h post-inoculation of either the P.C. or blood. n = 30−60 embryos. (C) Bacteria were enumerated from embryos at the indicated times post-inoculation of the P.C. (top) or blood (bottom). Each circle represents bacterial titers from individual embryos that were scored as live (open circles) or dead (shaded circles) prior to homogenization. The dashed line indicates the LOQ.

**Figure 1A** shows the dose- and niche-dependent killing of zebrafish embryos by the UPEC strains UTI89 and CFT073, the ABU isolate 83972, and the gut isolate HS, presented as Kaplan-Meier survival curves. Points on the curves only appear when at least one event (death) is recorded. Both high and medium inoculation doses of UTI89 and CFT073 were lethal after inoculation into the pericardial cavity, but only CFT073 was effective at killing the host when inoculated into the blood. In contrast, the ABU isolate 83972 was only lethal at high inoculation doses when delivered into the pericardial cavity, and had little effect when injected into the blood. HS was similarly avirulent when injected into the blood, but displayed moderate levels of lethality within the pericardial cavity. Interestingly, HS possesses many virulence-associated genes and has previously been suggested to act as a “precursor” pathogen [19].

**Figure 1B** summarizes results from all ten tested *E. coli* strains 24 h after inoculation of either the pericardial cavity or blood with ∼5,000 CFU. The laboratory K12 strains MG1655 and W3110 were the least virulent of the tested strains, causing no death unless exceptionally high inoculation doses of >30,000 CFU were used. Among the ExPEC strains, only the UTI isolates CFT073 and F11 were lethal in both the pericardial cavity and blood, while 536 and UTI89 caused significant host death only when inoculated into the pericardial cavity. In comparison to these UPEC strains, APEC01 and the NMEC isolate RS218 showed moderate levels of killing only after injection into the pericardial cavity, similar to gut isolate HS. Of note, all bacterial strains used in this study grew at similar rates in M9 medium at 28.5°C, the temperature at which zebrafish are maintained. Consequently, differences in virulence levels observed among these strains are likely not directly attributable to temperature effects on the rate of bacterial replication.

By microscopy, we observed that massive bacterial growth within either the pericardial cavity or blood was associated with eventual death of the zebrafish host. In all cases, the relatively clear pericardial cavity seen in uninfected or mock-infected embryos (**Video S3**) became increasingly cloudy following inoculation with lethal isolates such as CFT073 (**Video S4**). For these and later imaging studies, the *E. coli* strains used were transformed with the low-copy, high-retention plasmid pGEN-GFP(LVA), which encodes a constitutively expressed destabilized variant of GFP having a half-life of ∼40 min. The pGEN plasmid backbone contains the *hok sok* post-segregation killing system and two *par* loci that allow for high levels of plasmid retention over multiple generations in the absence of antibiotic selection [40],[41]. Use of the pGEN-GFP(LVA) construct facilitated the unambiguous detection of bacteria that were recently viable and translationally active. We observed that CFT073 and other lethal isolates eventually filled the entire pericardial cavity, but rarely spread into other niches until after the host had died (**Video S4**). In contrast, inoculation of either CFT073 or F11 into the blood resulted in the rapid spread and disseminated growth of these pathogens throughout the host circulatory system and, eventually, other tissues (**Video S5**). During the course of non-lethal infections, bacteria inoculated into either the pericardial cavity or blood failed to replicate appreciably and instead survived in only limited numbers within the host.

These trends were validated by determining bacterial titers in zebrafish homogenates at 0, 3, 6, 12, and 24 h post-inoculation of UTI89, CFT073, or MG1655 into the pericardial cavity or blood (**Figure 1C**; see below). In these assays, lethal infections were associated with increases in bacterial burden by up to 4 orders of magnitude, while during non-lethal infections bacterial numbers declined to near or below the limit of quantitation (LOQ). Interestingly, embryos that were challenged via the pericardial cavity with F11, UTI89, APEC01, RS218, 83972, and, less frequently, MG1655 and survived appeared at times to be notably sick, displaying restricted blood flow, heart deformation, edema, and/or curled tails, despite the absence of large numbers of bacteria (**Video S6**). These pathologies likely arise as a consequence of both host inflammatory responses and bacterial toxicity. In total, our data demonstrate that zebrafish embryos are relatively resistant to non-pathogenic laboratory *E. coli* strains, but are susceptible to ExPEC infection in a strain-, dose-, and niche-specific fashion.

### ExPEC genetic diversity and virulence in zebrafish

**Figure 2:**
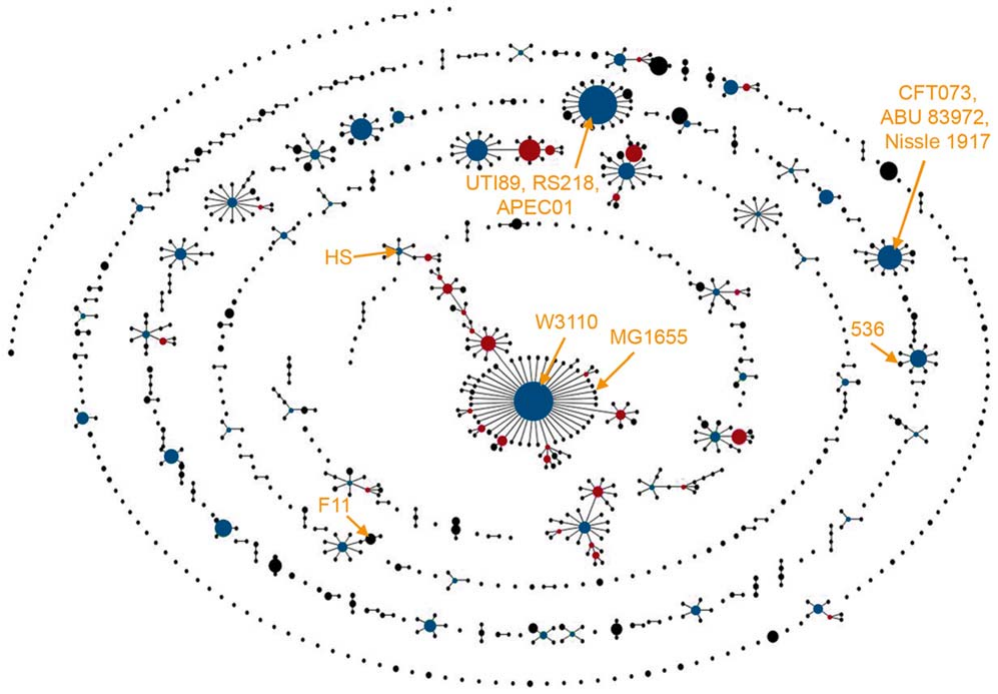
eBURST diagram of *E. coli*. The eBURST algorithm was used to generate a clustering diagram from 2,208 strains from the *E. coli* MLST database that is maintained by University College Cork. Circles represent single allele profiles (strain types, ST), based on seven genes (*adk*, *fumC*, *gyrB*, *icd*, *mdh*, *purA*, and *recA*). Size of each circle is proportional to the number of isolates that match a given allele profile. Blue circles are predicted founder genotypes, while red circles represent sub-founder genotypes. Solid lines radiating from founders and sub-founders denote single locus variants. Positions of the 10 sequenced *E. coli* isolates used in this study are indicated. Bootstrap confidence values for the founder groups associated with the highlighted strains are each 100%, except for the groups containing F11 and HS, which had values of 99% and 93%, respectively.

The genetic relatedness of the strains used in this study to one another and to the broader worldwide *E. coli* community was addressed using multilocus sequence typing (MLST) and the eBURST algorithm [42],[43], by which the allelic profiles of 2,208 *E. coli* isolates were compared and organized based on similarities among seven housekeeping genes (*adk*, *fumC*, *gyrB*, *icd*, *mdh*, *purA*, and *recA*) (**Figure 2**). For this analysis, isolates with identical allelic profiles were grouped together as strain types (circles) that were further organized into clonal clusters. These are defined as groups of genotypes that have six out of seven alleles in common with a founder genotype located in the center of each cluster (blue circles in Figure 2). This form of allelic-based cluster analysis is useful for identifying patterns of descent within bacterial populations in which horizontal gene transfer is common [44]. A similar approach was employed recently to infer relationships among bacteremic ExPEC and other *E. coli* isolates [45], but here we wished to assess if the genetic relatedness of the specific bacterial strains used in our assays could be correlated with the varying levels of virulence observed in the zebrafish host.

As shown in Figure 2, many of the *E. coli* strains used in this study possess the founder genotype of their respective clonal clusters and several share identical strain type profiles. Of note, the avian pathogen APEC01 and the human cystitis isolate UTI89, which were previously shown to have highly homologous genomes [5],[20], were grouped together as the same strain type in our analysis. Despite this similarity, APEC01 differed markedly from UTI89 in its virulence capacity within the pericardial cavity (p<0.001), and APEC01 instead appeared to be phenotypically akin to the more distantly related gut isolate HS (p>0.38; Figure 1B). Likewise, the pyelonephritis isolate CFT073 and ABU 83972 belong to the same clonal cluster, and yet display distinct levels of virulence in both the pericardial cavity and blood. Interestingly, current evidence indicates that some ABU isolates like 83972 were once pathogens, rendered less virulent by the loss or mutation of key virulence factors by genomic decay [46]. In total, our data confirm the idea that even pathogens with highly similar epidemiological and genetic origins can vary greatly in their virulence potential and niche-adaptability, probably due in part to acquisition of specific virulence factors by horizontal gene transfer.

### ExPEC toxins and bacterial virulence potential within zebrafish

**Figure 3:**
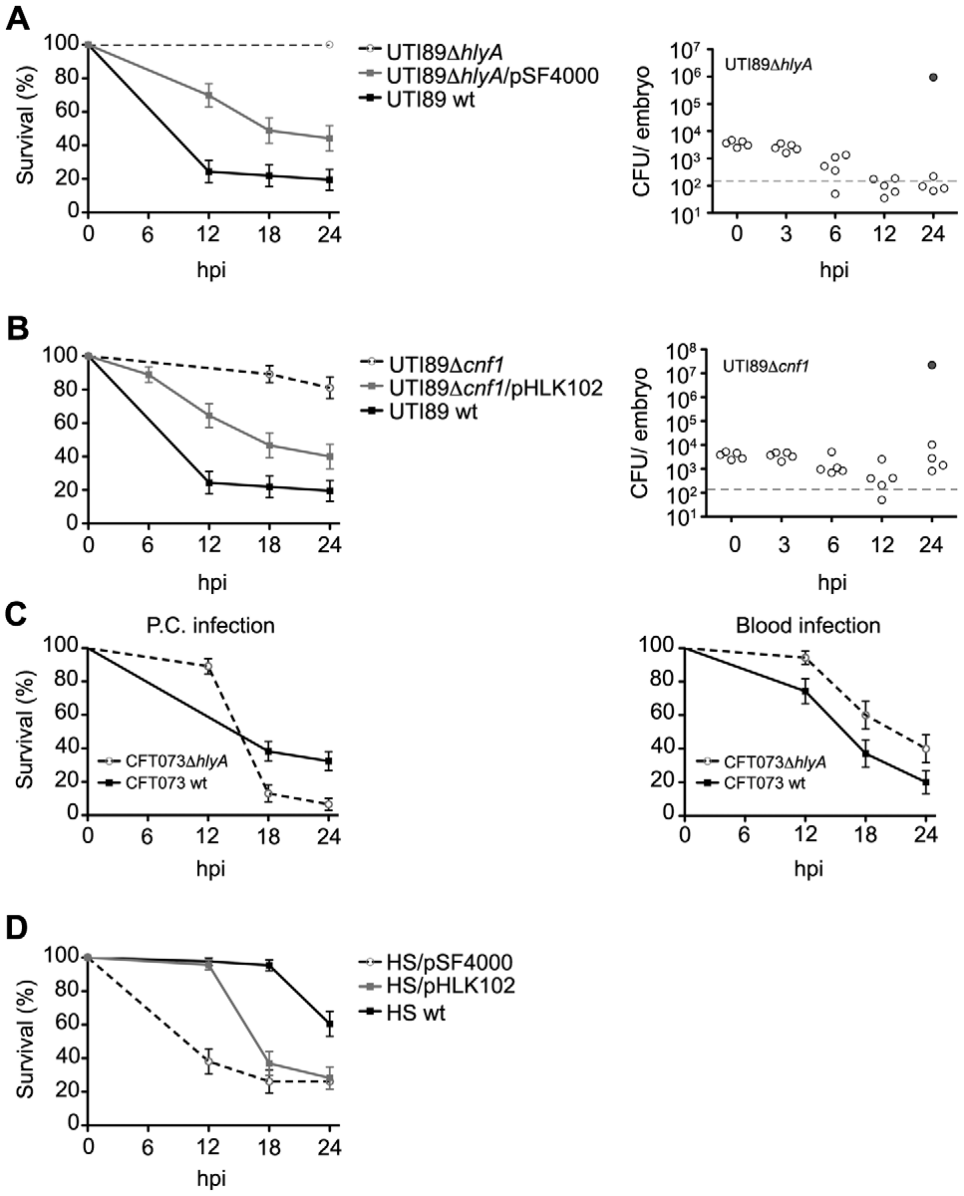
Differential effects of ExPEC-encoded toxins on zebrafish survival. (A) and (B), left panels - Zebrafish viability was determined at 0, 6, 12, 18, and 24 hpi of the P.C. with medium doses (4,000–6,500 CFU) of wild type (wt) UTI89, UTI89Δ*hlyA*, or UTI89Δ*hlyA*/pSF4000 (+Hly), UTI89Δ*cnf1*, or UTI89Δ*cnf1*/pHLK102 (+CNF1). Right panels - Bacterial burden was determined for UTI89Δ*hlyA* and UTI89Δ*cnf1* at the indicated hpi. Embryos were scored as live (open circles) or dead (shaded circles) prior to homogenization. The dashed line denotes the LOQ. (C) Zebrafish survival following inoculation of the P.C. (left) or blood (right) with wt CFT073 or CFT073Δ*hlyA*. Inoculum sizes used were 2,000–3,500 CFU for P.C. injections and 4,000–6,500 CFU for blood injections. (D) Zebrafish survival after P.C. inoculation with 4,000–6,500 CFU of wt HS (Hly- and CNF1-negative), HS/pSF4000 (+Hly), or HS/pHLK102 (+CNF1). Survival plots (A–D) are presented as Kaplan-Meier survival plots with standard error calculated by the Greenwood method. n = 30−60 embryos for all results.

We next asked if ExPEC virulence within zebrafish could be linked to known ExPEC-associated virulence factors, such as HlyA and CNF1. The pore-forming toxin HlyA is encoded by ∼50% of all ExPEC isolates, including strains UTI89, F11, CFT073, 536, and RS218 used here [47],[48]. Three of these isolates, UTI89, F11, and RS218, also encode CNF1, which is genetically linked with the *hly* operon in some ExPEC-associated PAIs [11],[49],[50]. In rodent infection models, both HlyA and CNF1 have been shown to influence ExPEC virulence, but the effects of either of these toxins on pathogen growth and persistence within the host have been more difficult to assess (data not shown and [21],[22],[23],[24],[51],[52],[53],[54]). We found that deletion of either *hlyA* or *cnf1* in UTI89 clearly attenuated both the virulence and growth capacity of this strain within the pericardial cavity of 48 hpf zebrafish embryos using medium inoculation doses of 4,000 to 6,500 CFU (**Figure 3A and B**, see below). Virulence was partially restored when either mutant strain was complemented with a plasmid carrying the respective deleted toxin. Surprisingly, disruption of *hlyA* in CFT073, which naturally lacks *cnf1*, did not diminish CFT073 virulence within the pericardial cavity and had only a slight attenuating effect in the blood (**Figure 3C**). This is despite the fact that the HlyA toxin encoded by CFT073 is nearly identical to the one expressed by UTI89.

The contribution of both HlyA and CNF1 to bacterial virulence in the zebrafish host was further addressed by transforming the gut isolate HS with plasmid pSF4000 or pHLK102, encoding the *hlyCABD* operon and *cnf1*, respectively. Expression of either toxin significantly enhanced the virulence of HS within the pericardial cavity (**Figure 3D**). Importantly, transformation of HS with control vectors (pSF4000 Δ*Bam*H1 or pBluescript II SK(-)) did not affect HS virulence (data not shown). These results build upon earlier work showing that fecal isolates transformed with plasmids encoding the *hlyCABD* operon can display substantially enhanced levels of cytotoxicity and virulence in cell culture and rat sepsis models [52],[53],[55]. In total, data from our experiments using recombinant UTI89 and HS strains conclusively show that the epidemiologically linked ExPEC–associated toxins HlyA and CNF1 can significantly impact not only bacterial virulence, but also bacterial persistence and multiplication within the host and, consequently, the eventual outcome of infection. However, our observations with CFT073 indicate that these toxins are dispensable in some genetic backgrounds and corroborates our previous supposition that ExPEC strains have likely evolved multiple and redundant virulence strategies for enhancing bacterial survival and growth within the host.

### ExPEC evasion of phagocyte-mediated killing

**Figure 4:**
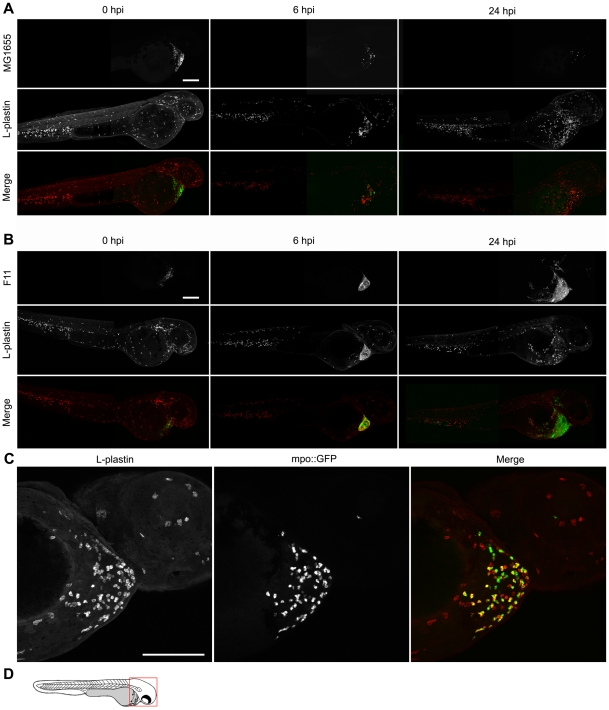
Phagocyte recruitment and abatement within the zebrafish pericardial cavity. (A and B) Zebrafish embryos were inoculated via the P.C. with 4,000–6,500 CFU of (A) the K12 strain MG1655 or (B) the ExPEC isolate F11, both carrying pGEN-GFP(LVA) for constitutive expression of destabilized GFP protein (green). Samples were fixed at 0, 6, and 24 hpi and processed for fluorescent confocal microscopy. Phagocytes (red) were detected using L-plastin-specific antibody. For clarity, the merged image at each time point is accompanied by images showing only corresponding single channel signals from bacteria or L-plastin. Each embryo was visualized by stitching together two z-projections generated from 40 to 50 5-µm-thick optical sections. Scale bar = 200 µm. (C) Tg(mpo::GFP) zebrafish embryos, in which GFP expression is under control of the neutrophil-specific MPO promoter, were injected via the P.C. with ∼5,000 CFU of the ExPEC isolate F11. At 4 hpi, samples were fixed and stained using anti-L-plastin antibody to label total phagocytes (red) relative to the GFP-positive neutrophils (green plus red). Bacteria present within the P.C. are not shown. Scale bar = 100 µm. (D) Diagram of a 48 hpf embryo with the area imaged in (C) highlighted by a red box. Embryos shown here are representative. All fish were viable at the time of sacrifice, except the 24 hpi F11-infected fish, which died prior to collection.

In mammals, phagocytes make up an essential frontline defense against many bacterial infections including those caused by ExPEC [36],[56],[57]. Zebrafish embryos at 48 hpf have a potent set of innate defenses, including both neutrophils and macrophages [35]. By immunofluorescence microscopy, we observed robust recruitment of these phagocytes into the pericardial cavity within 6 h post-inoculation of any of the *E. coli* strains tested (**Figures 4A and B**). Injection of PBS alone had no effect (data not shown). In these assays, *E. coli* strains carrying the low-copy, high-retention plasmid pGEN-GFP(LVA) were used for easy detection of viable, or recently viable, bacteria. Macrophages and neutrophils were visualized using an anti-L-plastin antibody that specifically labels these phagocytic cells [58]. In related experiments, the co-recruitment of both macrophages and neutrophils to sites of infection was verified using transgenic zebrafish embryos (Tg(mpo::GFP), [59]) that express GFP under control of a neutrophil-specific myeloperoxidase (MPO) promoter (for example, see **Figure 4C**).

**Figure 5:**
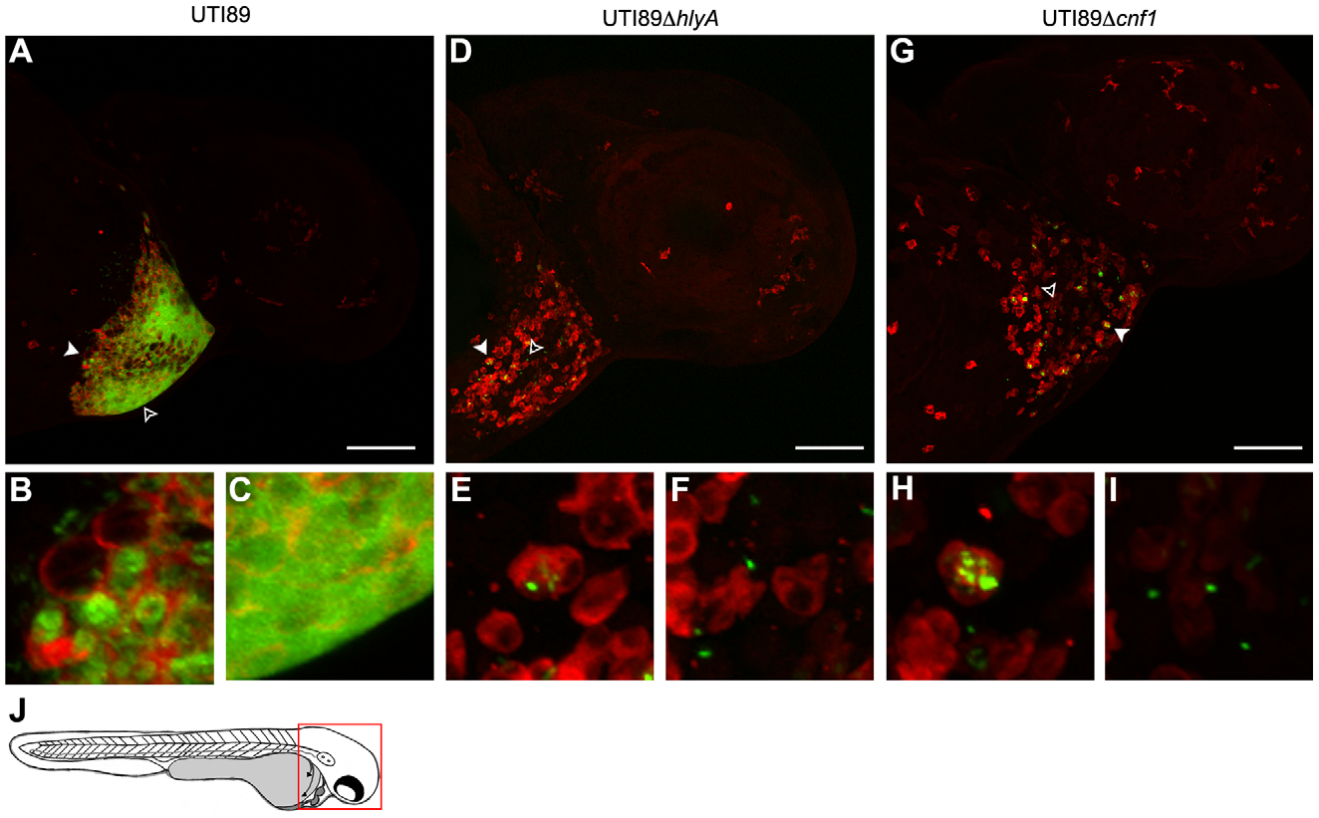
Phagocyte localization and bacterial internalization within the pericardial cavity. Zebrafish embryos were injected via the P.C. with 4,000–6,500 CFU of (A–C) wt UTI89, (D–F) UTI89Δ*hlyA*, or (G–I) UTI89Δ*cnf1*, each carrying pGEN-GFP(LVA) for constitutive expression of destabilized GFP protein (green). At 6 hpi, samples were fixed and processed for fluorescent confocal microscopy, using anti-L-plastin antibody to label phagocytes (red). Examples of internalized (solid arrowheads) and free extracellular (hollow arrowheads) bacteria in (A, D, and G) are shown at higher magnification in (B, E, and H) and (C, F, and I), respectively. Representative images are shown. All fish were viable at the time of sacrifice. Scale bars = 100 µm. (J) Diagram of a 48 hpf embryo with the area imaged in (A), (D), and (G) highlighted by a red box.

Phagocyte infiltration into the pericardial cavity following inoculation with the avirulent laboratory-adapted K12 strain MG1655 coincided with a sizeable reduction in bacterial numbers, as determined by confocal microscopy (**Figure 4A**). Despite equivalent or even higher chemotactic responses, phagocytes failed to eradicate the more lethal ExPEC isolates like F11, CFT073, 536, and UTI89 within the pericardial cavity, and instead were eventually overrun by the rapidly multiplying pathogens (as exemplified by F11 and UTI89, **Figures 4B** **and** **5A**). By 6 h post-inoculation, these bacteria were found in high numbers both within phagocytes and free within the pericardial cavity, often forming dense aggregates (**Figure 5A–C**). In contrast to wild type UTI89, the toxin-deficient mutants UTI89Δ*hlyA* and UTI89Δ*cnf1* were found in only low numbers at the same time point, typically within or closely associated with infiltrating phagocytes (**Figure 5D–I**). Notably, the rates and levels of phagocyte recruitment into the pericardial cavity, as assessed by microscopic observations, were similar following inoculation with wild type UTI89 or either of the two toxin mutants.

**Figure 6:**
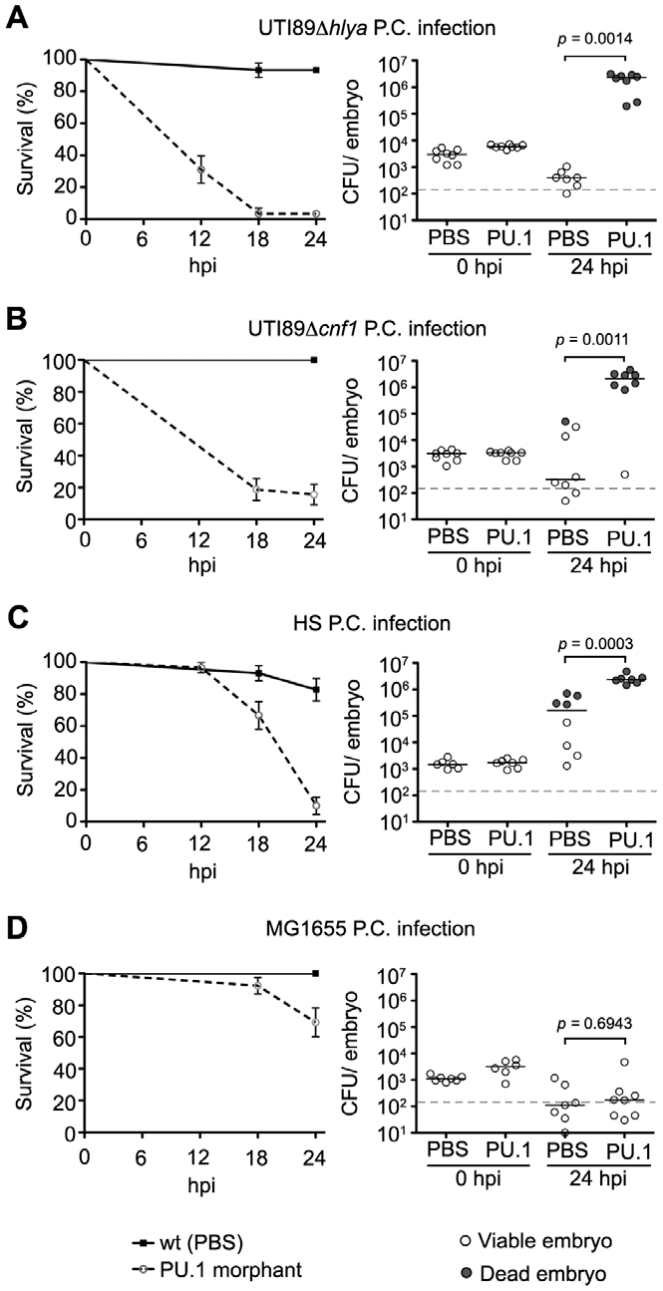
Zebrafish phagocytes are required for resolution of normally non-lethal pericardial *E. coli* infections. PU.1 morphants or normal PBS-injected control embryos were inoculated via the P.C. with 4,000–6,500 CFU of (A) UTI89Δ*hlyA*, (B) UTI89Δ*cnf1*, (C), HS or (D) MG1655. Host survival (left) was evaluated at 0, 6, 12, 18, and 24 hpi and is presented as Kaplan-Meier survival plots with standard error calculated by the Greenwood method. n = 30−60 embryos. (right) Scatter plots show levels of bacterial burden at 0 and 24 hpi in individual embryos that were scored as live (open circles) or dead (shaded circles) at time of collection. The horizontal bars represent median values for each group and the dashed line indicates the LOQ. The *P* values were determined by Mann-Whitney two-tailed analysis.

These findings suggest key roles for both HlyA and CNF1 in the evasion of phagocyte-mediated killing by ExPEC at local sites of infection. To further address this possibility, we used a translation-blocking morpholino to silence expression of the host PU.1 transcription factor, which drives the differentiation of myelopoetic precursors into macrophages and neutrophils [60]. Ablation of phagocyte development was confirmed using immunofluorescence microscopy of 48 to 72 hpf PU.1 morphants, by which substantial reduction in L-plastin staining was evident when compared with control embryos that were injected with PBS alone (data not shown). Following inoculation of the pericardial cavity, both UTI89Δ*hlyA* and UTI89Δ*cnf1* were significantly more lethal and grew to much higher titers in the PU.1 morphants relative to the PBS-injected control embryos (**Figure 6A and B**). Likewise, the gut isolate HS also displayed substantially increased lethality and growth within PU.1 morphants (**Figure 6C**). In contrast, the K12 strain MG1655 exhibited only slightly elevated virulence and no enhanced replication within the PU.1 morphants (**Figure 6D**). Presumably, other innate defenses and/or nutrient restrictions within the zebrafish host are sufficient to limit MG1655 survival in the absence, or near absence, of phagocytes.

**Figure 7:**
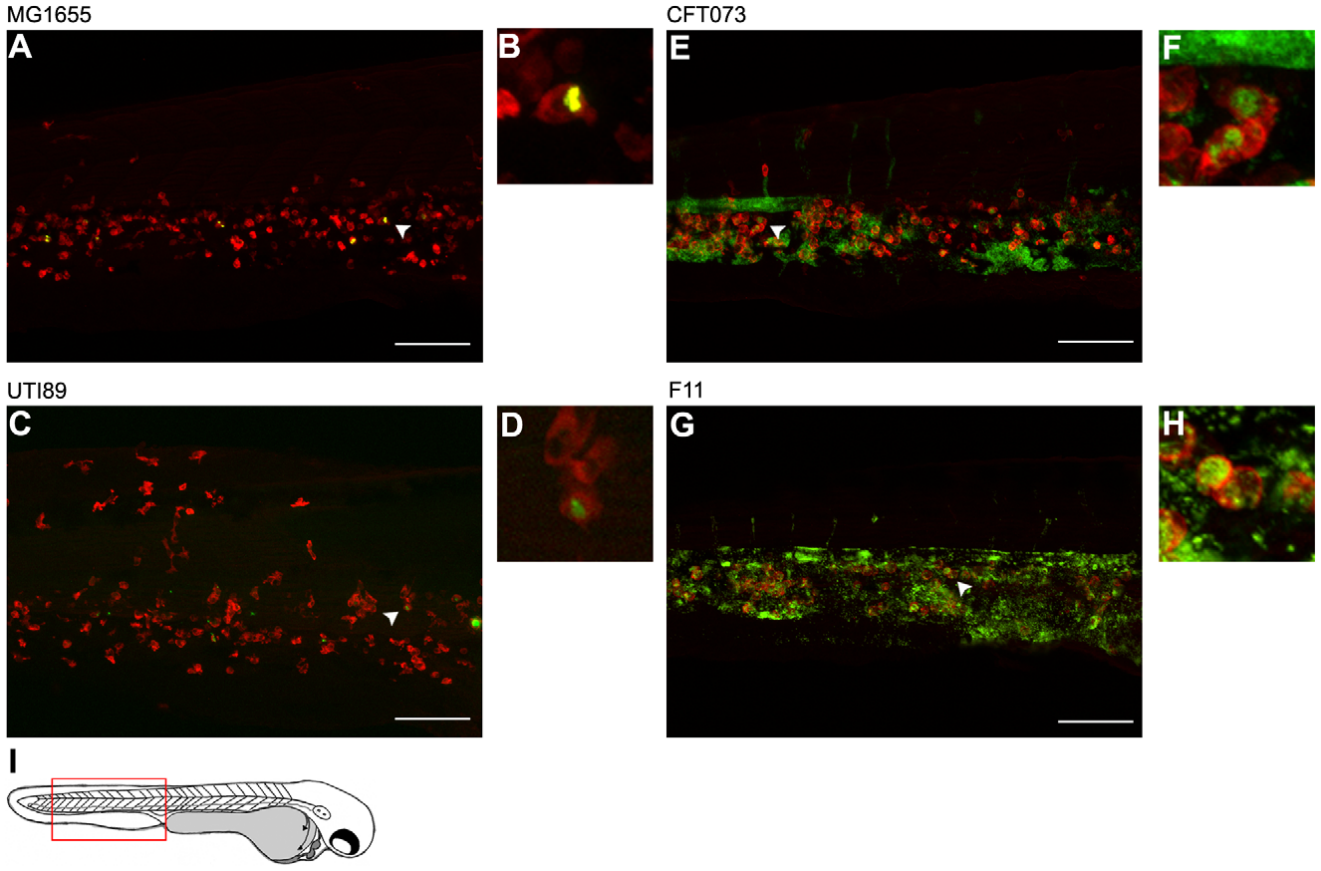
Differential growth and phagocytosis of *E. coli* isolates within the blood. Zebrafish embryos were infected via the blood with 4,000–6,500 CFU of (A and B) MG1655, (C and D) UTI89, (E and F) CFT073, or (G and H) F11. All bacterial strains carry pGEN-GFP(LVA) for constitutive expression of destabilized GFP (green). At 12 hpi, samples were fixed and phagocytes (red) were labeled using L-plastin-specific antibody for visualization by fluorescent confocal microscopy. Regions highlighted by arrowheads in (A), (C), (E), and (G) are shown further magnified in panels (B), (D), (F), and (H), respectively. All images shown are representative of the pool of embryos imaged. MG1655- and UTI89-infected embryos were viable and healthy in appearance prior to sacrifice for microscopy, whereas fish inoculated with CFT073 or F11 were notably sick and near death at time of collection. Scale bars = 100 µm. (I) Diagram of a 48 hpf embryo with the region imaged in (A), (C), (E), and (G) denoted by a red box.

**Figure 8:**
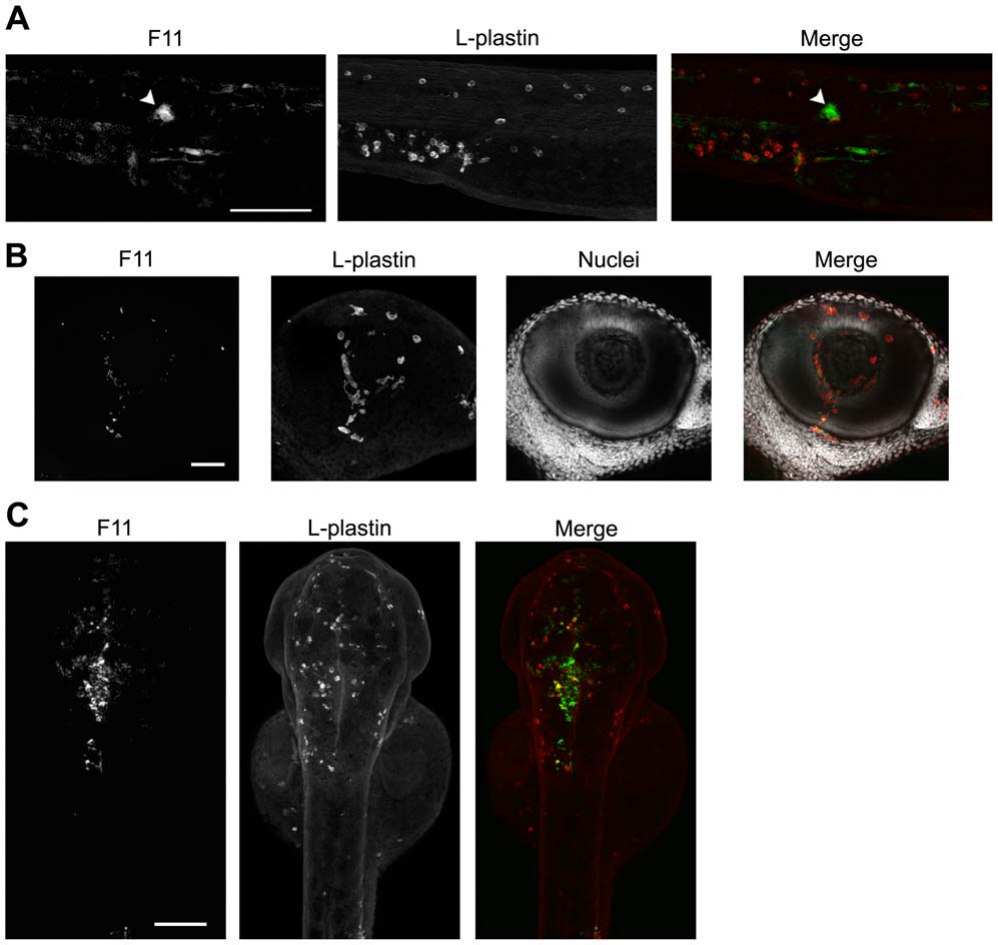
ExPEC isolates capable of blood colonization can disseminate and persist in a variety of host microenvironments. F11, carrying pGEN-GFP(LVA) for constitutive expression of destabilized GFP (green), was inoculated into the blood using a dose of 4,000–6,500 CFU. Samples were fixed at 6 or 12 hpi and phagocytes (red) were labeled using L-plastin-specific antibody for visualization by fluorescent confocal microscopy. (A) 20X z-projection of the tail region from an F11-infected embryo at 12 hpi. The arrowhead indicates a commonly observed bacterial microcluster. (B) 40X z-projection of the eye from an embryo at 6 hpi with F11. Hoechst nuclear dye (grey) was used to highlight the anatomical structure of the eye. (C) Dorsal to ventral 10X z-projection of the head region of an embryo at 12 hpi with F11. Scale bars = 100 µm for (A) and (C) and 50 µm for (B).

Not unexpectedly, phagocytes also appeared to be major facilitators of bacterial clearance within the blood and other niches outside of the pericardial cavity. By 12 h post-inoculation via the circulation valley, bacterial strains like MG1655 and UTI89 (which are for the most part non-lethal within the blood during the course of these assays, see Figure 1) were observed only in small numbers, primarily localized within phagocytes (**Figure 7A–D**). In contrast, and despite the presence of large numbers of circulating phagocytes, CFT073 and F11 multiplied to high levels within the blood and infiltrated multiple tissues before eventually killing the host (**Figure 7E–H**). In these assays, replication of CFT073 was typically restricted to the blood until late time points (see Video S5) while F11 was prone to disseminate into more diverse niches early on, often before growth of F11 in the blood was readily observable. For example, F11 frequently formed small groups, or “microclusters”, within tissues adjacent to blood vessels (**Figure 8A**). Interestingly, phagocytes were only occasionally seen in close association with the microsclusters, suggesting that these bacterial aggregates might be either physically inaccessible to phagocytes and/or actively interfering with phagocyte chemotaxis. Following inoculation via the circulation valley, F11 was also able to enter tissues within the eye (**Figure 8B**) as well as the brain (**Figure 8C** **and Video S7**), where phagocyte association with bacteria was limited.

**Figure 9:**
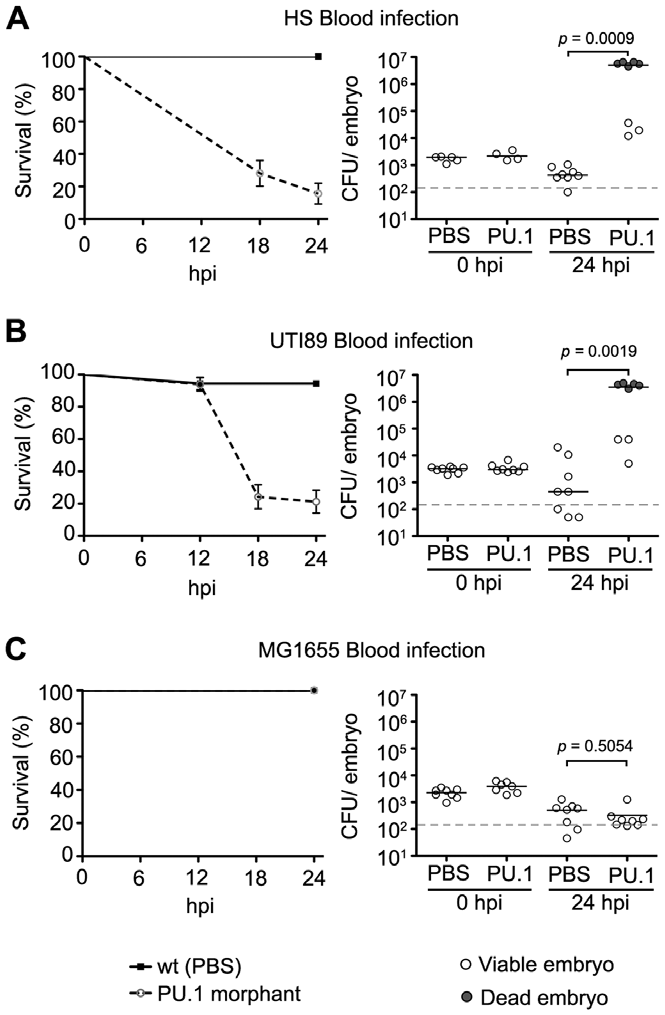
Zebrafish phagocytes are required for resolution of normally non-lethal *E. coli* infections in the blood. PU.1 morphants or normal PBS-injected control embryos were inoculated via the circulation valley with 4,000–6,500 CFU of (A) HS, (B) UTI89, or (C) MG1655. Host survival (left) was evaluated at 0, 6, 12, 18, and 24 hpi and is presented as Kaplan-Meier survival plots with standard error calculated by the Greenwood method. n = 30−60 embryos. Bacterial burden within individual embryos at 0 and 24 hpi is presented as scatter plots on the right. Embryos were scored as live (open circles) or dead (shaded circles) prior to collection. The horizontal lines indicate median values for each group while the dashed line indicates the LOQ. *P* values were calculated using Mann-Whitney two-tailed analysis.

To test the functional necessity of phagocytes in the control of systemic blood-borne *E. coli* infections within the zebrafish host, we again utilized PU.1 morphants as described above. Following inoculation via the circulation valley, the virulence and growth of both HS and the cystitis isolate UTI89 were significantly increased in PU.1 morphants relative to controls (**Figure 9A and B**). However, the virulence capacity and survival of MG1655 within the blood was not affected by ablation of phagocyte development (**Figure 9C**), mirroring results obtained after inoculation of PU.1 morphants via the pericardial cavity. In total, with the exception of MG1655, these results confirm an essential role for phagocytes in the control of both localized and systemic *E. coli* infections and indicate a functional link between the HlyA and CNF1 toxins and phagocyte evasion by ExPEC.

## Discussion

Classification of *E. coli* strains has traditionally relied upon serotype analyses based on the expression of specific O (somatic), K (capsular polysaccharide), and H (flagellar) antigens. Using such categorization schemes, some salient features of ExPEC isolates have been noted. For example, UPEC strains often elaborate only a certain subset of possible O antigens, which consist of 10–25 repeating sugar subunits linked to the outer core of lipopolysaccharide within the bacterial outer membrane [14],[61]. However, a functional role in pathogenesis for the UPEC-associated O antigens, which include O1, O2, O4, O6, O7, O8, O16, O18, O25, and O75, remains ambiguous. Additional gene-level characterization of ExPEC and related strains is advancing with the development of more rapid and inexpensive genome sequencing approaches, along with the use of comparative genomic hybridization and related array technologies [16],[19],[20],[45],[62],[63],[64],[65],[66],[67],[68]. While these approaches can supply valuable information regarding the prevalence, relatedness, and expression profiles of known and putative ExPEC virulence factors, these methods on their own cannot definitively indicate a functional role for specific bacterial genes in pathogenesis. Results presented here outline the utility of a novel zebrafish infection model as a means to resolve ExPEC virulence mechanisms, demonstrating that genotypically closely related ExPEC isolates can vary significantly *in vivo* in their pathogenic properties as well as their dependency upon specific virulence factors.

In our zebrafish infection assays, we compared seven sequenced ExPEC isolates with two lab-adapted K12 strains and the gut isolate HS. The K12 strains were avirulent except when exceptionally high inoculation doses were used, while all tested ExPEC isolates, with the exception of ABU 83972, were virulent to a significant degree when between 4,000 and 6,500 CFU were injected into the pericardial cavity. The blood presented a much more restrictive environment in these 24-hour assays, where only the cystitis isolate F11 and the pyelonephritis isolate CFT073 were able to replicate and eventually kill the host. The diverse levels of niche-adaptability and virulence displayed by the ExPEC strains in zebrafish embryos were not strictly correlated with the epidemiological characteristics or source of each isolate. For example, within the blood the virulence capacities of the two human cystitis isolates UTI89 and F11 did not match at all, and instead UTI89 virulence levels were more similar to the pyelonephritis isolate 536 (see Figure 1B). Likewise, strain serotypes were not necessarily reflective of strain virulence potential. F11 and 536, for example, both display the O6 antigen (see Table 1), but only F11 was lethal when inoculated into the blood. However, F11 and CFT073 both share the K2 capsular antigen, which may contribute to their virulent phenotypes in both the blood and pericardial cavity. Notably, in mouse UTI models the K2 capsule of CFT073, as well as the K15 capsule of strain 536, has been shown to enhance bacterial fitness and virulence, providing evidence that capsular antigens can influence the outcome of infection [69],[70].

**Table 1:** Bacterial Strains and Plasmids.

Strain or Plasmid	Description	Source or Reference
*E. coli*		
F11	UPEC (cystitis isolate, O6:K2*:H31)	[16]
UTI89	UPEC (cystitis isolate, O18:K1:H7)	[66],[84]
536	UPEC (pyelonephritis isolate, O6:K15:H31)	[64]
CFT073	UPEC (pyelonephritis isolate, O6:K2:H1)	[71]
RS218	NMEC (meningitis isolate, O18ac:K1:H7)	[85],[86]
APEC01	APEC (avian sepsis isolate, O1:K1:H7)	[5]
ABU 83972	Probiotic (ABU isolate, OR:K5:H-)	[87],[88]
HS	Commensal (gut isolate, O9)	[19],[89]
MG1655	K12 lab strain	[90]
W3110	K12 lab strain	[91]
*Salmonella*		
TT23691	Template strain with Kan^r^ cassette flanked by universal primer sequences	John Roth, [81]
Plasmids		
pKM208	Encodes IPTG-inducible lambda Red recombinase; Amp^r^	[83]
pSF4000	*hlyCABD* in pACYC184; Clm^r^	[79]
pHLK102	*cnf1* in pBluescript II SK(−) (pHLK102); Amp^r^	[21]
pSF4000 Δ*Bam*H1	*hlyABD* Δ*hylC*, control for pSF4000; Clm^r^	[79]
pBluescript II SK(−)	Empty vector control for pHLK102; Amp^r^	Clontech
pGEN-luxCDABE	Parental plasmid used for construction of pGEN-GFP(LVA), contains *hok sok* post-segregation killing system and two *par* loci; Amp^r^	[40],[41]
pGEN-GFP(LVA)	Encodes destabilized GFP (half-life ∼40 minutes) under control of the *em7* promoter; Amp^r^	This study
Recombinant strains		
UTI89/pKM208	UTI89 with pKM208	[81]
UTI89*ΔhlyA*	UTI89 *hlyA::kan*	This study
UTI89*ΔhlyA*/pSF4000	UTI89 *hlyA::kan* complemented with pSF4000	This study
UTI89*Δcnf1*	UTI89 *cnf1::clm*	[12]
UTI89*Δcnf1*/pHLK102	UTI89 *cnf1::clm* complemented with pHLK102	This study
CFT073/pKM208	CFT073 with pKM208	This study
CFT073*ΔhlyA*	CFT073 *hlyA::kan*	This study
HS/pSF4000	HS with pSF4000	This study
HS/pHLK102	HS with pHLK102	This study
HS/pSF4000 Δ*Bam*H1	HS with pSF4000 Δ*Bam*H1	This study
HS/pBluescript II SK(−)	HS with pBluescript II SK(−)	This study

*: Presence of the K2 capsule is inferred from the available genomic sequence of F11.

Evolutionary relationships among the tested ExPEC isolates, based on clonal clusters determined by eBURST [43], were also weak predictors of the virulence potential and adaptability displayed by each tested strain. Specifically, strains within the same clonal group, implying similar phylogeny, did not necessarily have comparable virulence attributes. This likely reflects the mosaic architecture of ExPEC genomes, which results from the incorporation of foreign genetic elements by horizontal gene transfer [20],[71],[72]. The acquisition of these accessory genes can presumably impart distinct niche-specific growth and survival advantages, causing even very closely related bacteria to diverge with respect to their virulence potential.

The transformative power of gene acquisition and loss by ExPEC and related *E. coli* isolates was highlighted by our work with *hlyA* and *cnf1*. Disruption of either of these genes severely attenuated the virulence and growth of UTI89 within the pericardial cavity of zebrafish embryos (see Figure 3). In contrast, CFT073 naturally lacks *cnf1* and the targeted deletion of *hlyA* had little effect on the virulent nature of this pathogen within either the pericardial cavity or the blood. The introduction of plasmids encoding either the *hlyCABD* operon or *cnf1* into HS converted this gut isolate from a modestly virulent strain into a much more pathogenic isolate. This finding is particularly intriguing considering previous observations that HS and other commensal isolates, such as Nissle 1917, already possess many virulence-associated genes and may only require the acquisition or modification of one or more additional factors to become full-fledged pathogens [19],[63]. Our results with HS, along with earlier work using the fecal isolate J198 [53], indicate that this transformation is feasible with the addition of just a single virulence factor, supporting the notion that intermingling and genetic exchange among commensals and ExPEC isolates within the gut can lead to the generation of new pathogens.

These observations raise the questions of how *cnf1* and *hlyA* promote UTI89 and HS survival and virulence within the host and why these factors are dispensable to CFT073. For strains like UTI89, at least, it appears that *hlyA* and *cnf1* may enable this pathogen to neutralize phagocytes that are drawn to sites of infection. In our zebrafish infection model, we observed robust recruitment of both macrophages and neutrophils into the pericardial cavity as well as to bacterial loci that develop elsewhere during systemic infections. Within the pericardial cavity, UTI89 persisted and grew to high numbers despite massive phagocyte infiltration. By confocal microscopy, these phagocytes often appeared distended and frequently contained large numbers of viable, or at least recently viable, bacteria (see Figure 5). The presence of so many wild type GFP-positive bacteria within phagocytes may be a consequence of highly active phagocytosis during the course of our infection assays, but may also reflect an innate ability of some ExPEC isolates to persist and perhaps even multiply with these effector immune cells. This possibility requires further investigation. In sharp contrast to wild type UTI89, the *hlyA* and *cnf1* UTI89 mutants failed to grow within the pericardial cavity and instead were typically found only in small clusters within the infiltrating phagocytes.

Work in mouse and cell culture model systems has demonstrated that high levels of HlyA can cause osmotic lysis of host cells, while sublytic concentrations of this pore-forming toxin can modulate host survival pathways and thereby interfere with phagocyte chemotaxis and bactericidal activities [12],[73],[74],[75],[76]. By causing the aberrant activation of host Rho-family GTPases, CNF1 can similarly inhibit phagocyte functions [11],[77],[78]. Both HlyA and CNF1 may also stimulate the breakdown of tissue barriers and the release of host nutrients [24],[51],[79], but in our assays phagocytes appear to be the primary targets of these toxins. This conclusion is strongly supported by our results showing that UTI89*ΔhlyA*, UTI89*Δcnf1*, and wt HS (without either *hlyA* or *cnf1*) are lethal in PU.1 morphants that lack both neutrophils and macrophages (see Figures 6 and 9). Depletion of these host immune cells also substantially enhanced the virulence of wild type UTI89 when injected into the blood, suggesting that phagocytes are the major obstacles to the growth of ExPEC strains like UTI89 during systemic infections.

The differential requirements for *hlyA* and *cnf1* observed in UTI89 versus CFT073 indicate a degree of functional redundancy in the spectrum of virulence factors that ExPEC strains can acquire. Specifically, our data indicate that CFT073 must have alternate mechanisms to neutralize or overwhelm host phagocytes, independent of HlyA or CNF1. It is likely that the disparate degrees of virulence observed among the tested ExPEC isolates are also influenced by differences in expression levels and/or allelic variation of toxins like HlyA, as has been documented previously [52],[53]. However, this cannot completely account for the differential requirements that strains like CFT073, F11, and UTI89 have for HlyA and CNF1. Specifically, CFT073 remains highly virulent even in the absence of *hlyA* and ongoing work in our lab suggests the same is true for F11. In addition, *in vitro* levels of hemolytic activity do not necessarily correlate with the virulence capacity of individual strains. For example, we recently found that the pyelonephritis isolate CP9 exhibits high levels of hemolytic activity on blood agar plates at both 28.5 and 37°C, similar to CFT073 and F11, and yet CP9 is substantially less virulent in the zebrafish host (unpublished observations).

In conclusion, development of a zebrafish infection model has allowed us to perform side-by-side phenotypic comparisons of the virulence potentials and niche restrictions of multiple ExPEC isolates and mutant strains, something that has rarely been undertaken with any group of bacterial pathogens. Although much of this study focused on phenotypes associated with *hlyA* and *cnf1*, ongoing work has begun to identify additional gene products as key facilitators of ExPEC colonization and virulence within the zebrafish host. These include four regulators of ExPEC virulence previously identified in mouse UTI models: the RNA chaperone Hfq and three secretin proteins (HofQ, YheF, and GspD) associated with putative type IV pili and type II secretion systems (unpublished data and [80],[81]). Ultimately, the amenability of the zebrafish infection model to high-throughput screens and real-time imaging, as well as its ability to resolve both host and bacterial factors that contribute to ExPEC virulence and growth during both systemic and localized infections, may facilitate the development of more efficacious therapies for the prevention and treatment of ExPEC-mediated illnesses.

## Materials and Methods

### Ethics statement

Zebrafish used in this study were handled in accordance with IACUC-approved protocols following standard procedures (www.zfin.org).

### Bacterial strains and plasmids

All bacterial strains and plasmids used in this study are listed in Table 1. Bacteria were grown static at 37°C for 24 h in 20 ml M9 minimal medium (6 g/l Na_2_HPO_4_, 3 g/l KH_2_PO_4_, 1 g/l NH_4_Cl, 0.5 g/l NaCl, 1 mM MgSO_4_, 0.1 mM CaCl_2_, 0.1% glucose, 0.0025% nicotinic acid, 0.2% casein amino acids, and 16.5 µg/ml thiamine in H_2_O). Antibiotics (chloramphenicol, kanamycin, or ampicillin) were added to the growth medium when necessary to maintain recombinant plasmids. Targeted knockout of *hlyA* was created in UTI89 and CFT073 using the lambda Red-mediated linear transformation system [82],[83]. Briefly, a kanamycin resistance cassette with 40 base pair overhangs specific for the target *hlyA* gene in UPEC was amplified from the chromosome of *Salmonella* strain TT23691 using the primers MP104 (5′-GTAGTGTGCTTTTAATTTGTGCAGTGGTTATTGTTGGCATCACCAAACACCCCCCAAAACC-3′) and MP105 (5′-CATATGGACGGAACTCAATAACTTTGACAGCATCAGCA TACACACAACCACACCACACCAC-3′). PCR products were introduced by electroporation into UTI89 or CFT073 carrying pKM208, which encodes IPTG (isopropyl-β-D-thiogalactopyranoside)-inducible lambda Red recombinase. Disruption of *hlyA* was verified by PCR and growth on blood agar plates at 37°C, where clear zones developed around only hemolysin-positive colonies. Of note, the hemolytic activity of the tested wild type ExPEC isolates did not change noticeably on blood agar plates grown at 37°C versus 28.5°C.

The plasmid pGFP(LVA) (Clontech) was used as a template for PCR amplification of *gfp(LVA)*, with added 5′-SnaBI and 3′-NcoI restriction sites, using primers MP126 (TGATACGTATGCAGGTCGACTCTAGAGGAT) and MP127 (TGACCATGGACGGC CGACTAGTAGGTCA). The PCR product was digested and ligated into SnaBI and NcoI sites of pGEN-luxCDABE (kindly provided by H. Mobley, University of Michigan Medical School, [41]), behind the constitutive synthetic promoter *em7*. The resulting construct, pGEN-GFP(LVA), is retained in the absence of antibiotic selection over many bacterial generations due to the presence of the *hok sok* post-segregation killing system and two *par* (partitioning) loci [40].

### Zebrafish embryos

*AB wild-type or recombinant Tg(mpo::GFP) zebrafish embryos were collected from a laboratory-breeding colony that was maintained on a 14-h/10-h light/dark cycle. Embryos were grown at 28.5°C in E3 media (5 mM NaCl, 0.17 mM KCl, 0.4 mM CaCl_2_, 0.16 mM MgSO_4_) containing 0.000016% methylene blue as an anti-fungal agent.

### Generation of PU.1 morphants

A morpholino specific for knockdown of PU.1 (5′-GATATACTGATACTCCAT TGGTGGT-3′) has been described previously [60] and was purchased from Gene Tools, LCC. Approximately 1 nL of a 500 µM working concentration of the morpholino in phosphate buffered saline (PBS) was injected into one- to two-cell stage embryos. Control embryos were injected in parallel with 1 nl of sterile PBS alone. PU.1 morphants between 48 and 72 hpf were verified by reduction in L-plastin (a pan leukocyte marker [58]) staining, as determined by immunofluorescence microscopy.

### Infection of zebrafish embryos

One ml from each 24 h bacterial culture was pelleted, washed once with 1 ml sterile PBS (Hyclone), and re-suspended in ∼275–550 µl PBS to obtain appropriate bacterial densities for microinjection. Prior to injection, 48 hpf embryos were manually dechorionated, briefly anesthetized with 0.77 mM ethyl 3-aminobenzoate methanesulfonate salt (tricaine) (Sigma-Aldrich), and embedded in 0.8% low melt agarose (MO BIO Laboratories) without tricaine. Approximately 0.5, 1.0, or 2 nl of bacteria were injected directly into the pericardial cavity or the blood via the circulation valley located ventral to the yolk sac using a YOU-1 micromanipulator (Narishige), a Narishige IM-200 microinjector, and a JUN-AIR model 3-compressor setup. For each experiment, average CFU introduced per injection were determined by adding 10 drops of each inoculum into 1 ml 0.7% NaCl, which was then serially diluted and plated on Luria-Bertani (LB) agar plates.

After injection, embryos that were pericardially infected were left embedded in agar and submerged in E3 media without tricaine or methylene blue. Embryos that were injected in the blood were carefully extracted from the agar and placed individually into wells of a 96-well microtiter plate (Nunc) containing E3 media lacking both tricaine and methylene blue. Fish were examined every 6 h over the course of a 24 h period and scored for “death”, defined here as the complete absence of heart rhythm and blood flow. Survival graphs depict total pooled results from two or more independent experiments in which groups of 15 to 20 embryos were injected. To quantify bacterial numbers during the course of infection, embryos were homogenized at the indicated time points in 500 µL PBS containing 0.5% Triton X-100 using a mechanical PRO 250 homogenizer (PRO Scientific). Homogenates were then serially diluted and plated on MacConkey agar (Sigma-Aldrich). *E. coli* selectively grow as pink colonies on this medium and thus are easily discerned from occasional bacterial contaminants from fish water that form white colonies.

### Statistical analysis

Kaplan-Meier survival and scatter plots were generated using GraphPad Prism 5. For Kaplan-Meier survival plots, standard error was calculated using the Greenwood method and the log-rank (Mantel-Cox) test was used to determine statistical differences between data sets. Two-tailed Mann-Whitney statistical analyses were performed on scatter plots to determine significant differences between medians. *P* values of less than 0.05 are considered significant.

### Microscopy

Zebrafish embryos were injected with either PBS or ∼5,000 CFU of the indicated bacterial strain, each carrying pGEN-GFP(LVA). At 0, 6, 12, and 24 h post-inoculation, embryos were fixed by rotating overnight at 4°C in PBS containing 4% paraformaldehyde and 0.4% Triton X-100. Embryos were then washed 3×5 min at room temperature in wash buffer (PBS containing 0.8% Triton X-100), placed in blocking buffer (PBS plus 0.25% casein, 0.1% Tween, and 1% dimethyl sulfoxide) for 2 h, and then labeled with rabbit anti-L-plastin antibody (1∶5000, [58]) overnight at 4°C with rotation. The embryos were again washed 4×20 min at room temperature prior to addition of donkey anti-rabbit Alexa555 fluor-conjugated secondary antibody (1∶1000; Invitrogen) in blocking buffer. After rocking overnight at 4°C and subsequent washes, the embryos were rinsed 3X in 30%, 50%, and 80% glycerol/PBS solutions. Samples were then stored in 80% glycerol at −20°C or directly mounted in 80% glycerol and imaged using an Olympus IX81 FV1000 confocal microscope equipped with 10X, 20X, and 40X objectives. Confocal stacks were assembled using ImageJ (National Institutes of Health) and subsequently stitched together using Photoshop CS3 (Adobe Systems Inc.). Live injection and infection videos were obtained using a Canon PowerShot A640 10 megapixel camera mounted on an SZX10 stereomicroscope (Olympus) using a Camadapter kit (camadapter.com).

### Cluster analysis of *E. coli*

Allelic profiles from 2,208 *E. coli* isolates were downloaded from the MLST database at the Environmental Research Institute (ERI, University College Cork, http://mlst.ucc.ie). The eBURST algorithm (http://eburst.mlst.net/) was used with 1,000 bootstrap iterations to organize strains into clonal clusters and to generate an eBURST diagram [43].

## Supporting Information

Figure S1 Schematic of 48 hpf zebrafish anatomical features. Microinjection sites used in this study are indicated by shaded arrowheads. Definitions: P.C., pericardial cavity; circ. val., circulation valley; C.V., caudal vein; Nc, notochord; D.A., dorsal aorta.
(0.19 MB TIF)

Video S1 The movie depicts the inoculation of the pericardial cavity of a 48 hpf zebrafish embryo with approximately 10,000 CFU of MG1655/pGEN-GFP(LVA).
(3.48 MB MOV)

Video S2 The movie shows about 10,000 CFU of MG1655/pGEN-GFP(LVA) being injected into the blood of a 48 hpf zebrafish embryo via the circulation valley.
(2.93 MB MOV)

Video S3 Movie depicts healthy uninfected 48 hpf embryos using dark field and oblique light, as well as fluorescent (green) as a control. Note the clarity of the pericardial cavity, heart structure, heartbeat, and blood flow in comparison to Movies S4, S5 and S6 taken using ExPEC-infected embryos.
(4.20 MB MOV)

Video S4 Zebrafish embryo infected with CFT073/pGEN-GFP(LVA) via the pericardial. Clips show state of the pericardial cavity at 6, 12, and 24 hpi.
(5.79 MB MOV)

Video S5 Zebrafish embryo infected with CFT073/pGEN-GFP(LVA) via the circulation valley. Clips show bacterial dissemination throughout the circulation at 12 hpi. By 18-24 hpi most CFT073-infected fish will have died and bacteria become dispersed throughout most tissues.
(10.64 MB MOV)

Video S6 Embryos that survive challenge with F11, UTI89, APEC01, RS218, 83972, and, less often, MG1655 may still appear notably sick, despite the absence of large numbers of bacteria. Clips show examples of restricted blood flow, heart deformation, edema, and curled tails in embryos at various time points post inoculation of the pericardial cavity with UTI89 or APEC01.
(7.43 MB MOV)

Video S7 Movie shows sequential merged 10X z-stacks of the F11/pGEN-GFP(LVA)-infected embryo depicted in Figure 8C. Phagocytes labeled with anti-L-plastin antibodies are shown in red.
(1.80 MB MOV)
