# Supplementary figures and images for: Use of Zebrafish to Probe the Divergent Virulence Potentials and Toxin Requirements of Extraintestinal Pathogenic Escherichia coli
Source: PLoS Pathog. 2009 Dec 18;5(12):e1000697. doi: 10.1371/journal.ppat.1000697 (PMC2785880; doi:10.1371/journal.ppat.1000697)

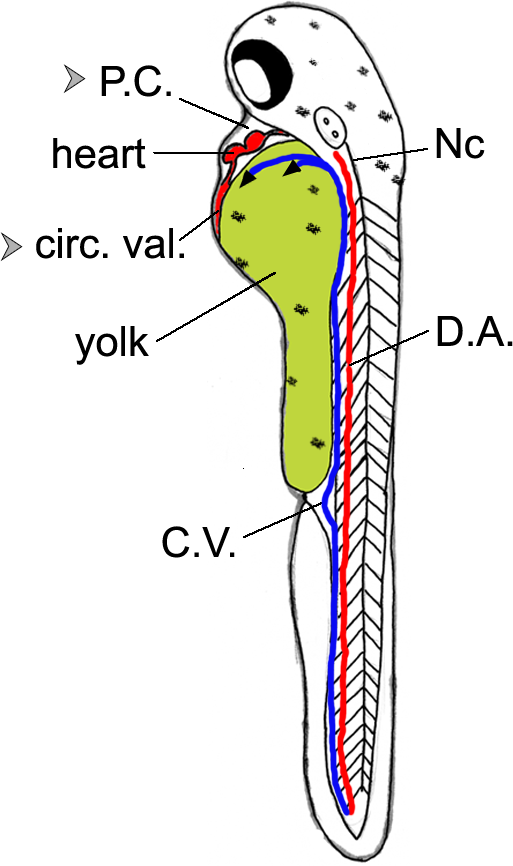

Supplement: Figure S1 — Schematic of 48 hpf zebrafish anatomical features. Microinjection sites used in this study are indicated by shaded arrowheads. Definitions: P.C., pericardial cavity; circ. val., circulation valley; C.V., caudal vein; Nc, notochord; D.A., dorsal aorta. (0.19 MB TIF) [file ppat.1000697.s001.tif]
